# Climate-Driven Futures of Olive (*Olea europaea* L.): Machine Learning-Based Ensemble Species Distribution Modelling of Northward Shifts Under Aridity Stress

**DOI:** 10.3390/plants14243774

**Published:** 2025-12-11

**Authors:** Muhammed Mustafa Özdel, Beyza Ustaoğlu, İsa Cürebal

**Affiliations:** 1 Independent Researcher, Kayseri 38050, Türkiye; m.mustafaozdel@gmail.com; 2 Department of Geography, Faculty of Humanities and Social Sciences, Sakarya University, Sakarya 54050, Türkiye; 3 Department of Geography, Faculty of Art and Science, Balıkesir University, Balıkesir 10145, Türkiye; curebal@balikesir.edu.tr

**Keywords:** olive (*Olea europaea* L.), species distribution modelling (SDM), ensemble modelling, aridity index (UNEP AI), sustainable agriculture

## Abstract

With its millennia-long agricultural history, Olive (*Olea europaea* L.) is one of the most strategic crops of the Mediterranean basin and a key component of the Turkish economy. This study assessed the effects of climate change on the potential distribution of olive in Türkiye using machine learning-based species distribution models (SDMs). Analyses were conducted using the 1970–2000 reference period and future projections for 2041–2060 and 2081–2100 under the SSP2-–4.5 and SSP5–8.5 scenarios, incorporating bioclimatic variables as well as topographic factors such as elevation, slope, and aspect. The model showed strong predictive performance (AUC = 0.93; TSS = 0.77) and identified elevation, winter precipitation (Bio19), and mean temperature of driest quarter (Bio9) as the primary variables influencing the distribution of olive trees. Model results predict a significant shift in suitable areas for olive cultivation, both northward—from the traditional Aegean and Mediterranean coastal belt toward the Marmara and Black Sea regions—and upward in elevation into higher-altitude inland areas. High-suitability areas, which accounted for 4.4% of Türkiye’s land area during the reference period, are projected to decline to 0.2% by the end of the century under the SSP5–8.5 scenario. UNEP Aridity Index analyses indicate increasing aridity pressure on olive habitats. While 87.2% of suitable habitats were classified as sub-humid in the reference period, projections for 2081–2100 under SSP5–8.5 suggest that 40.1% of these areas will shift to dry sub-humid and 26.4% to semi-arid conditions.

## 1. Introduction

**Figure 1:**
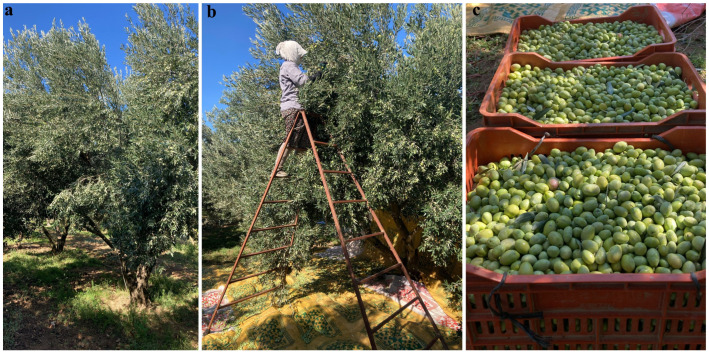
Olive harvesting conducted in a major olive-producing region of western Türkiye (Aydın): (**a**) General view of olive trees. (**b**) A traditional harvesting method using a comb, in which the fruits fall onto sheets spread beneath the trees. (**c**) After harvesting, the collected olives are stored in crates and transported to processing facilities for further operations.

The olive (*Olea europaea* L.), with its millennia-long agricultural history, is one of the most important crops of the Mediterranean basin and has played a central role in the nutrition, culture, and trade of Mediterranean civilizations for centuries [1]. Today, it remains economically significant for producing countries due to its diverse uses in industries such as edible oil, table olives, health products, cosmetics, and food. According to average production data for 2014–2023, Spain, Greece, Italy, Türkiye, and Morocco are among the leading olive-producing countries [2]. Türkiye ranks among the world’s leading countries in both olive production volume and product diversity, with olive cultivation holding strategic importance for the national economy, rural development, and cultural heritage. The country’s diverse climate types and geomorphological features, even over short distances, result in significant regional variations in olive production. According to average production data for 2015–2024, the Aegean (50%), Mediterranean (24%), Marmara (20%), and Southeastern Anatolia (5%) regions are the main contributors, whereas Central Anatolia, the Black Sea, and Eastern Anatolia regions account for much smaller shares [3]. Despite the presence of modern plantation areas nationwide, olive groves cultivated using traditional methods still represent a substantial portion of total production (Figure 1).

The reality of climate change has been documented as a global phenomenon [4,5,6,7], and its impacts have been extensively demonstrated in studies worldwide [8,9,10]. In recent years, the Mediterranean basin has experienced significant temperature increases and more frequent heatwaves, making it a key region for observing climate change effects [11]. In addition to temperature changes, irregular precipitation patterns are reinforcing aridity trends in the region [12,13,14,15]. Due to its geographical location, Türkiye is directly affected by these trends, with aridity conditions particularly prevalent in the southern, central, and western regions [16,17,18]. Future projections indicate that ariditys may occur more frequently and with greater intensity [19,20]. On the other hand, some national-level studies in Türkiye highlight changes in temperature and precipitation patterns, as well as the increasing frequency of extreme weather events. These studies indicate that certain regions are particularly vulnerable to climatic impacts, which could result in significant environmental and socio-economic challenges [21,22,23].

Agriculture is highly dependent on atmospheric conditions, and changing climate patterns are placing significant pressure on agricultural production [24]. Rising temperatures and aridity particularly affect rain-fed agriculture, where irrigation opportunities are limited or infrastructure is inadequate. The depletion of water resources can threaten production sustainability even in modern plantation areas with sufficient irrigation systems. For climate-sensitive crops, physiological needs may not be met [25], yield losses may occur [26,27], and the prevalence of pests and diseases may increase [28]. Consequently, producers may shift to more resilient or low-water-requirement crops to maintain economic viability [29], potentially altering the geographical distribution of agricultural production areas [30]. Conversely, areas that were previously unsuitable for agriculture or had low yield potential may become suitable for production, or experience increased yields due to changes in environmental conditions. For example, Chen et al. [31] reported that rising temperatures led to increased cotton yields in northwestern China and the Yellow River basin, while yields declined in the Yangtze River valley in central and southern regions. Farooq et al. [32] indicated that wheat and maize production could rise at higher latitudes under climate change, whereas rice production may decline due to reduced water availability. Similarly, Adão et al. [33] suggested that the bioclimatic suitability of twelve Portuguese grape varieties is likely to shift from their traditional growing regions in Portugal and Italy to northern Spain, France, and higher-altitude areas under future climate scenarios.

The olive tree is an evergreen, long-lived species that exhibits a degree of aridity tolerance [34]. Its climatic resilience allows it to thrive in regions with hot, dry summers and mild winters. Additionally, its low soil selectivity [35] enables growth across diverse edaphic conditions, contributing to its wide ecological distribution. The species requires an annual average temperature of at least 14.5 °C, while temperatures below −7 °C are critical, and total annual rainfall of 700–850 mm is necessary for optimal growth [36]. Considering its current geographical distribution and cultivation requirements, the olive tree has specific ecological needs and is therefore vulnerable to changing climatic conditions. Rising temperatures, more frequent heatwaves, and prolonged aridity periods may alter the suitability of olive-growing areas [37,38,39], cause seasonal shifts in phenological stages [40,41], and lead to fluctuations in production patterns [42,43,44,45]. Environmental factors such as temperature, rainfall patterns, light duration, and water availability shape the phenological development of the olive tree, determining the timing of flowering, fruit set, and ripening stages. The olive tree also requires a specific chilling period for optimal development and consistent yield, as well as a minimum amount of rainfall under non-irrigated conditions. When sufficient chilling duration is not provided during the winter period, the tree’s flowering process may be delayed or become irregular [46]. Therefore, changes in phenological stages, along with abiotic stress factors such as temperature fluctuations, aridity, and soil moisture, play a decisive role in olive production, affecting both yield and product quality.

Species distribution models (SDMs) are a fundamental tool for assessing the effects of climate change on ecosystems and predicting the future distribution potential of species [47]. These models characterize species’ ecological niches and identify their geographically suitable areas by relating observed distribution data to environmental variables [48]. In recent years, statistical and machine learning-based algorithms have been widely employed in SDM studies due to their effectiveness in capturing the relationships between species and environmental factors [49,50,51,52,53,54,55]. More recently, ensemble modelling approaches have gained prominence. By combining outputs from multiple algorithms, ensemble models reduce the biases and uncertainties inherent in individual models. This integration leverages the strengths of different methods, providing more reliable assessments of species’ potential responses under current and future climate conditions and offering greater scientific credibility for ecological decision-making processes [56].

This study aimed to determine the potential distribution areas of the olive tree (*Olea europaea* L.), a species of significant ecological and economic importance in Türkiye, under past climate conditions (1970–2000) and future projections (2041–2060 and 2081–2100) based on the SSP2–4.5 (moderate) and SSP5–8.5 (pessimistic) scenarios. An ensemble modelling approach was employed, combining statistical and machine learning-based methods with climate projections from multiple global circulation models (GCMs). By integrating outputs from different modelling methods (model ensemble) and multiple GCMs (GCM ensemble), uncertainties associated with individual models were reduced. Another key objective was to examine the relationship between future aridity conditions and olive habitat suitability. Future aridity analyses were conducted using the UNEP Aridity Index under the same periods and scenarios, also evaluated via the GCM-ensemble approach, allowing direct comparison of habitat suitability and aridity trends on the same climatic basis.

The sub-objectives of this research are structured around the following questions:

(i) Which environmental variables most influence the distribution of olive trees in the study area according to the models?
(ii) How will the spatial distribution of suitable areas for olive trees change under future climate scenarios compared to the 1970–2000 reference period?
(iii) Which regions are projected to experience losses in suitability, and which regions may become more favourable for cultivation?
(iv) Is the aridity tolerance of olive trees changing—that is, are olive trees likely to be exposed to drier conditions in the future?
(v) Is there a statistically significant relationship between aridity conditions and the suitability classes of olive trees?

## 2. Materials and Methods

### 2.1. Species Occurrence Data

**Figure 2:**
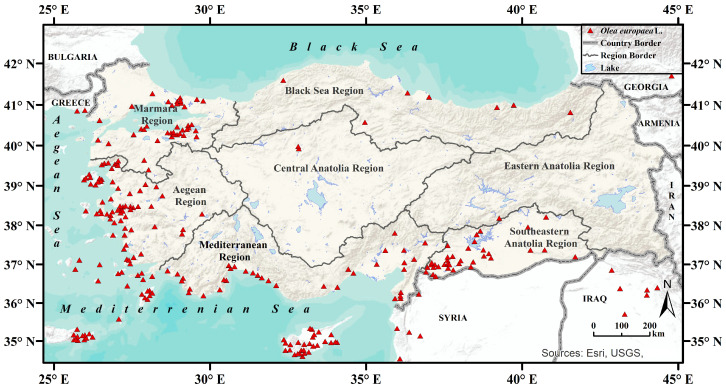
Occurrence records (n = 256) of olive (*Olea europaea* L.) used in the study.

Occurrence data for olive trees were compiled from the GBIF (Global Biodiversity Information Facility) database [57] and GPS coordinates collected during field observations conducted at various times and locations. Each GBIF record [57] was individually verified using Google Earth Pro to assess spatial accuracy, and records with questionable or incorrect locations were removed from the dataset. To prevent overfitting, a 5 km spatial filtering was applied to the occurrence records using the ‘Spatially Rarefy Occurrence Data’ tool in ArcMap (version 10.8.1) SDM Toolbox v2.5 [58,59]. After filtering, 256 records were retained for analysis (Figure 2). Absence data, representing areas unsuitable for olive cultivation, were randomly generated in the R environment. To reduce spatial autocorrelation, a 10,000 km^2^ spatial filtering was also applied to these absence records in R.

Occurrence records and environmental raster data were prepared to cover Türkiye and its neighbouring regions, as shown in Figure 2. Including neighbouring regions in the model helps reduce boundary effects and allows the model to capture ecological gradients more accurately. However, since the study focuses solely on the distribution of olive trees within Türkiye, the final suitability maps were clipped to the country’s borders. In addition, Tuz Lake, Van Lake, and large dam reservoirs were specifically excluded from the study area, as these aquatic systems do not provide realistic habitat conditions for olive trees, particularly in terms of hydrology and soil characteristics. These areas were masked from the model outputs to prevent false indications of suitability in future scenarios. The final maps and subsequent calculations were generated within this framework.

### 2.2. Environmental Layers Used in the Modelling Process

Bioclimatic variables are widely recognized as effective indicators in species distribution models, as they collectively capture environmental conditions related to temperature and precipitation [60]. These variables allow for a comprehensive assessment of both climatic constraints and the suitability of habitats for species. Temperature and precipitation, in particular, play a decisive role in the geographical distribution of olive trees, making bioclimatic variables essential for accurately evaluating their ecological requirements and distribution patterns in this study. In addition to climatic factors, environmental variables such as altitude, slope, and aspect also influence olive tree distribution. Altitude is especially important, as it affects temperature gradients, frost risk, evaporation and soil moisture, wind exposure, and sunlight duration, thereby shaping the species’ ecological tolerance limits. In Türkiye, olive cultivation is economically viable at altitudes of 0–800 m, and approximately 75% of olive groves are located on sloping terrain [34,36].

The study utilized 19 bioclimatic variables (Figure S1) derived from temperature and precipitation data, encompassing both historical data from 1970–2000 and future projections for 2041–2060 (near future) and 2081–2100 (distant future). These datasets were obtained from WorldClim v2.1 (https://www.worldclim.org/) at a spatial resolution of 30 s [61]. The 1970–2000 period was used as the reference to ensure comparability with future scenarios. For future projections, seven different global circulation models (BCC-CSM2-MR, CMCC-ESM2, HadGEM3-GC31-LL, INM-CM5-0, IPSL-CM6A-LR, MRI-ESM2-0, MIROC6) within the CMIP6 framework, capable of representing temperature, precipitation, and extreme climate events for Türkiye and its surroundings, were employed. SSP2–4.5 (moderate) and SSP5–8.5 (pessimistic) scenarios were combined using an ensemble approach to generate averaged climate projections. This method reduced uncertainties associated with relying on a single predictor and produced more comprehensive and reliable predictions by integrating multiple climate predictors [62]. In addition to bioclimatic variables, topographical factors such as elevation, slope and aspect were also used as independent variables in modelling species distribution; these factors were generated using 30 s resolution SRTM elevation data provided by WorldClim.

### 2.3. Multicollinearity Check and Variable Selection

In this study, Variance Inflation Factor (VIF) analysis was applied to 19 bioclimatic variables, along with elevation, slope, and aspect, to prevent multicollinearity issues. This method was chosen for its accuracy and reliability in assessing linear relationships and potential interactions among variables. As there is no consensus in the literature on an exact VIF threshold [63,64,65] and as a more precise selection criterion, a threshold of 5 was adopted, and variables exceeding this value—indicating potential multicollinearity—were removed from the model [66,67]. Subsequently, Pearson correlation analysis was applied to the remaining variables, confirming that strong linear relationships had been minimized and redundant information was reduced. The retained variables effectively capture the information content of the excluded variables due to their high correlations, thereby preserving the model’s predictive accuracy and spatial distribution [68].

### 2.4. Ensemble Modelling and Mapping

In this study, the R-based ‘sdm’ package was used to evaluate the effects of environmental variables on the distribution areas of olive trees, as it allows the integration of multiple modelling approaches [69]. This package enables the comparative application of various statistical and machine learning algorithms on a single platform and supports the creation of ensemble models by combining individually statistically determined models. Such ensemble models provide more balanced and generalizable predictions than individual models [70]. Thirteen algorithms available in the sdm package (version 1.2-59) in RStudio 2024.12.1 (R version 4.4.1) were applied to estimate both the reference period and future potential distribution of olive trees. The algorithms used were: Random Forest (RF), Maximum Entropy (MaxEnt), Boosted Regression Trees (BRT), Multivariate Adaptive Regression Splines (MARS), Generalized Additive Model (GAM), Support Vector Machines (SVM), Generalized Linear Models (GLM), GLMNET (Lasso and Elastic Net Regularization), Classification and Regression Trees (CART), Flexible Discriminant Analysis (FDA), Mahalanobis Distance (Mahal.dismo), Domain (Domain.dismo), and Bioclim [71].

These models were evaluated using the criteria widely accepted in species distribution modelling (SDM) studies, namely the area under the ROC curve (AUC) and the True Skill Statistic (TSS). AUC values range from 0.5 to 1, with values below 0.5 considered equivalent to random classification, 0.8–0.9 indicating good performance, and 0.9–1 representing excellent model performance [72]. While AUC reflects the overall discriminative power of a model, it does not provide information regarding threshold selection [73]. TSS, in contrast, is calculated at a specific threshold and incorporates sensitivity and specificity. TSS is obtained by subtracting 1 from their sum to account for both false positives and false negatives. TSS values range from −1 to +1, where +1 indicates perfect classification, 0 represents random prediction, and negative values indicate worse-than-random performance [74]. In the literature, TSS values are considered weak between 0.2 and 0.5, useful between 0.6 and 0.8, and excellent above 0.8 [75]. To ensure reliable and robust ensemble models, thresholds of AUC ≥ 0.9 and TSS ≥ 0.7 were applied, with models below these values excluded from further analysis. Model outputs were generated from the remaining models in 10 repetitions and then combined to create ensemble models. The dataset was partitioned into 80% for training and 20% for testing [51,76,77].

The continuous logistic outputs of the ensemble models range from 0 to 1, representing the probability of habitat suitability (*p*) for olive trees. For ease of visualization and interpretation, these values were classified into four categories using equal interval methods: unsuitable (*p* < 0.25), low (0.25 ≤ *p* < 0.5), moderate (0.5 ≤ *p* < 0.75), and high (*p* ≥ 0.75) [78,79]. The model results for the 1970–2000 reference period demonstrated substantial consistency with the current distribution of olive trees and production statistics reported in the literature, validating the chosen threshold classification [3,34,80,81]. Future projections were calculated in km^2^ and as percentages relative to the reference period model. Gain and loss maps were subsequently prepared for the SSP2–4.5 (2041–2060, 2081–2100) and SSP5–8.5 (2041–2060, 2081–2100) scenarios, with changes quantified through area calculations. These maps were obtained through change analysis performed by overlaying the reference period with future scenarios.

### 2.5. Analyses Related to Aridity

The United Nations Environment Programme (UNEP) Aridity Index (AI) was employed to assess the relationship between the potential distribution of olive trees and aridity under future climate scenarios. The UNEP AI is defined as the ratio of precipitation (P) to potential evapotranspiration (PET) (Equation (1)). PET was calculated using the Thornthwaite method (Equations (2)–(4)) [82]. In Equation (2), AdjF is a coefficient that adjusts PET based on the average day length in the month. Td represents the average daily temperature (°C) calculated for each month, with values below 0 °C set to 0 in the calculations. I is the annual heat index, derived from the twelve-month average temperatures (Tm), reflecting the total heat accumulated over the year. The parameter a is an empirical coefficient calculated from I, which adjusts the effect of temperature change on PET [83].

(1) $$\mathrm{UNEP}\;\mathrm{AI}=\frac{P}{\mathrm{PET}}$$

(2) $$\mathrm{PET}=16\times \mathrm{AdjF}\times {\left(\frac{10\times \mathrm{Td}}{I}\right)}^{a}$$

(3) $$I={\sum_{i=1}^{12}\left(\frac{\mathrm{Tm}}{5}\right)}^{1.514}$$

(4) $$a=(6.75\times 10^{-7})I^{3}-(7.71\times 10^{-5})I^{2}-(1.792\times 10^{-2})I+0.49239$$

The UNEP AI is a method endorsed by the United Nations (UN) and the Food and Agriculture Organization (FAO) in global-scale aridity and desertification studies and is widely used to characterize regional water scarcity [84,85,86]. In this study, UNEP AI was implemented in the R environment. The dataset comprised total precipitation and average temperature data corresponding to the SSP2–4.5 and SSP5–8.5 scenarios for the periods 1970–2000, 2041–2060, and 2081–2100. The models were classified in the ArcMap environment based on the classification scheme presented in Table 1 [83,87,88,89].

Various analyses were conducted in the R environment to examine the relationship between SDMs and aridity. First, the suitability classes (moderate suitable and highly suitable habitats) from the 1970–2000 reference period and future projections were compared with aridity indices, and the frequency distributions of these suitability classes across aridity categories were calculated as percentages. The frequency distribution revealed changes in the aridity tolerance of olive trees relative to the reference period, indicating whether areas classified as suitable were shifting toward drier conditions. To statistically quantify the contribution of aridity to habitat losses observed in the change models derived from suitability maps, Binomial Logistic Regression was applied to areas of loss and the corresponding aridity models [90].

In this approach, β coefficients indicate the effect of variables on the probability of habitat loss, *p*-values assess statistical significance, odds ratios (ORs) quantify the magnitude of effects in probability terms, Pseudo R^2^ reflects model explanatory power, and AUC measures classification success. Binomial Logistic Regression is a widely used method for examining relationships between categorical dependent variables and continuous or categorical independent variables [91]. This method allowed the statistical assessment of the extent to which aridity conditions influence olive habitat suitability in this study.

## 3. Results

### 3.1. Variable Selection

**Figure 3:**
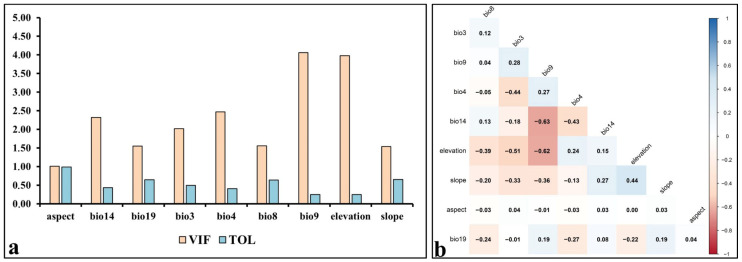
Analyses used to assess multicollinearity: (**a**) Results of the VIF analysis, (**b**) Results of the Pearson correlation analysis.

VIF (Variance Inflation Factor) and TOL (Tolerance) analyses were performed to address multicollinearity among environmental variables. The results indicated no multicollinearity among the nine retained variables, confirming their suitability for use in the modelling process (Figure 3a). Pearson correlation analysis, conducted to further verify the absence of multicollinearity, also showed no significant correlations between these variables (Figure 3b). Consequently, from the 22 environmental variables initially evaluated, 9 key variables were selected that could effectively represent the potential distribution of olive trees without introducing multicollinearity. These variables are: altitude, slope, aspect, Bio3 (Isothermality), Bio4 (Temperature Seasonality), Bio8 (Mean Temperature of the Wettest Quarter), Bio9 (Mean Temperature of the Driest Quarter), Bio14 (Precipitation of the Driest Month), and Bio19 (Precipitation of the Coldest Quarter).

### 3.2. Model Performance Evaluation

The 13 individual algorithms listed in Table 2 were initially applied in the modelling process using the selected environmental variables. A success threshold of AUC ≥ 0.9 and TSS ≥ 0.7 was established to minimize the influence of poorly performing algorithms that could reduce the predictive power of ensemble models. Ensemble modelling was then constructed using the high-performance algorithms that met these criteria: RF, MaxEnt, BRT, MARS, GAM, and SVM. Although the AUC and TSS values of these successful models were similar, RF, MaxEnt, and BRT exhibited the highest performance. The resulting ensemble model achieved an average AUC of 0.927 and a TSS of 0.768 (Table 2).

### 3.3. Contributions of the Variables

The evaluation based on the successful algorithms revealed that the contribution levels of environmental variables to the potential distribution of olive trees differed (Table 3). Among all variables, elevation exhibited the highest contribution and was particularly influential in the MARS, GAM, and MaxEnt models. Following elevation, the most significant variables were Bio19, Bio9, Bio4, and Bio8. The remaining variables had lower contribution levels, ranging from 0.01 to 0.03, and played a more limited role in shaping the model outputs.

### 3.4. Potential Distributions

#### 3.4.1. Distribution by Reference Period 1970–2000

**Figure 4:**
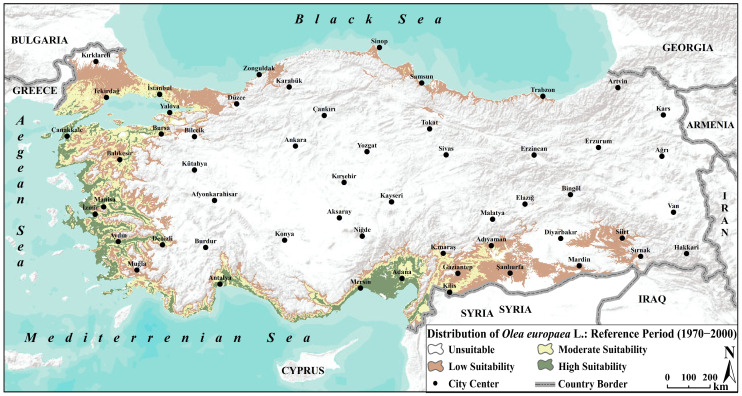
Ensemble species distribution modelling of olive (*Olea europaea* L.) for the reference period (1970–2000).

The model for the recent past, based on the 1970–2000 reference period, closely reflects the current distribution of olive trees in Türkiye. The model results indicate that areas of high suitability are primarily concentrated along the Aegean coast in the west and the Mediterranean coastline in the south. In addition, the southern part of the Marmara Region (around Çanakkale, Balıkesir, and Bursa), located in the northwest of the country and characterized by a transitional climate between Mediterranean and Black Sea/continental climates, also emerges as an important high-suitability area. This finding coincides with the reality of intensive and productive olive production in these regions, where the effects of the Mediterranean climate are evident. Moderately suitable areas form a transition zone extending from these highly suitable coastal regions towards the interior. Notably, moderate suitability is also observed in the western part of Southeastern Anatolia (around Gaziantep and Kilis), which experiences a Mediterranean-influenced climate disrupted by continental effects, placing conditions near the ecological tolerance limits of olive trees. In contrast, areas along the Black Sea coast in the north show low suitability, while the interior and eastern regions, characterized by high plateaus, are largely unsuitable for olive cultivation (Figure 4). The harsh continental climate conditions and severe winter frosts in these regions are the primary factors limiting olive cultivation. Area calculations indicate that approximately 80% of the country is unsuitable for olive distribution, whereas around 11% comprises areas of medium and high suitability (Table 4).

#### 3.4.2. Distributions Based on Future Projections

**Figure 5:**
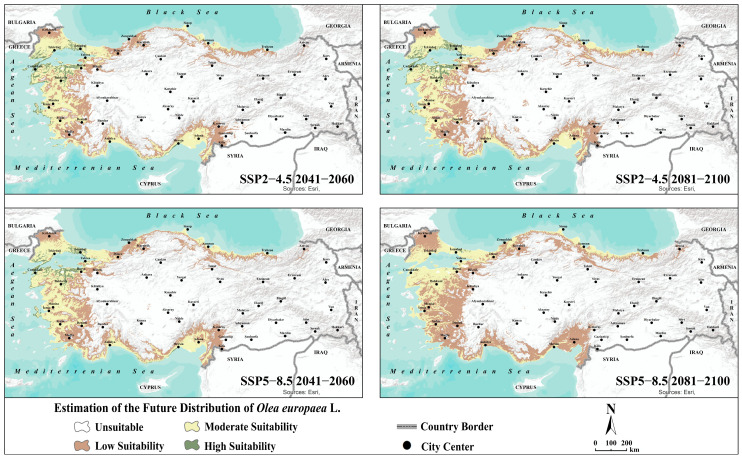
Ensemble species distribution modelling of olive (*Olea europaea* L.) under future climate scenarios.

Future projections indicate substantial changes in the potential distribution of olive trees in Türkiye under climate change scenarios. In both SSP2–4.5 and SSP5–8.5 scenarios, areas suitable for olive cultivation are generally shifting northward and toward higher-altitude inland regions. This shift reflects the declining suitability of coastal areas due to rising temperatures and increasing aridity stress. Traditional olive-growing regions that were highly suitable during the reference period, particularly along the Aegean and Mediterranean coasts, are projected to lose suitability in the near future (2041–2060), with these losses becoming even more pronounced in the distant future (2081–2100). Under the pessimistic SSP5–8.5 scenario, areas of high suitability along these coastal regions are predicted to almost completely disappear. Notable changes are also projected in southern inland areas, such as Adıyaman, Gaziantep, and Kilis, where medium and low suitability classes are projected to transition to unsuitable conditions, even under the moderate climate change scenario (Figure 5). Quantitative analyses support these spatial change: highly suitable areas, which accounted for 4.4% of the country’s land area in the reference period, are projected to decline to 2.3% in the near future and to 0.2% by the end of the century under SSP5–8.5 scenario (Table 4). Conversely, inland regions previously unsuitable for olive cultivation, along with portions of the Black Sea coastal belt, are becoming low- and moderately suitable. Consequently, low suitability areas are projected to increase significantly from 9.4% in the reference period to 15.7% by the end of the century, even under the most pessimistic scenario (Table 4).

### 3.5. Changes in Olive Suitability

**Figure 6:**
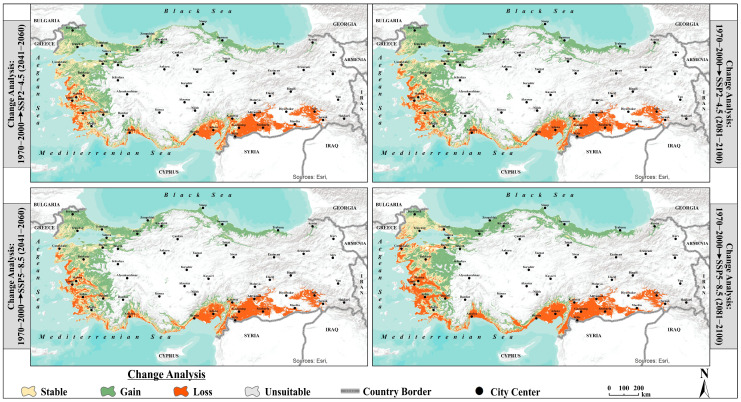
Projected changes in suitability classes of olive (*Olea europaea* L.) under SSP2−4.5 and SSP5−8.5 climate scenarios relative to the reference period.

The change analysis reveals a consistent trend across all scenarios: olive cultivation areas are shifting from southern coastal regions, such as the Mediterranean and South Aegean, toward cooler inland and northern areas, including the Inner Aegean, Marmara, and Black Sea regions. This shift results in significant losses of suitability in existing olive groves, while new areas previously unsuitable for olive cultivation gain suitability. Alongside these dynamic changes, stable areas—particularly in the Aegean and southern Marmara regions—emerge as potential future refuges for olive cultivation under climate change. These areas may serve as strategically important, resilient zones for sustaining future olive production, as the models indicate they can maintain their suitability despite changing climatic conditions (Figure 6).

Under the SSP2−4.5 scenario, losses are projected at 8% in the near future, increasing slightly to 9% in the distant future. In the SSP5−8.5 scenario, losses rise from 8.6% to 11.3% over the same periods, representing the highest projected loss. The proportion of gain areas increases over time in the SSP2−4.5 scenario, rising from 10.8% in the near future to 14.5% in the distant future. In contrast, under SSP5−8.5, the gain proportion is 12.6% in the near future, decreasing to 12.3% by the end of the century, indicating that gains are more widespread under moderate emission conditions. The proportion of stable areas remains limited in both scenarios and declines over time. In SSP2−4.5 scenario, stable areas decrease from 8.7% to 7.1%, while in SSP5−8.5 scenario, they decrease from 7.8% to 7%. These areas are scattered across the country and represent the regions with the lowest level of change. Conversely, unsuitable areas account for the largest proportions in all scenarios. Under SSP2−4.5, the proportion of unsuitable areas decreases slightly from 72.5% to 69.4%, and under SSP5−8.5, from 71% to 69.5% (Table 5).

### 3.6. The Relationship Between Suitable Areas and Aridity

Projections of future distribution of Türkiye’s climate classes based on the UNEP AI framework indicate a clear and increasing trend of aridification across the country. During the reference period of 1970–2000, dry sub-humid areas—representing transitional climates between semi-arid and semi-humid regions—were observed in Central Anatolia and Southeastern Anatolia, while distinctly humid climates occurred along the Black Sea coastal belt and in Eastern Anatolia. The semi-arid class was limited to a very small area near the Syrian and Armenian borders during this period.

**Figure 7:**
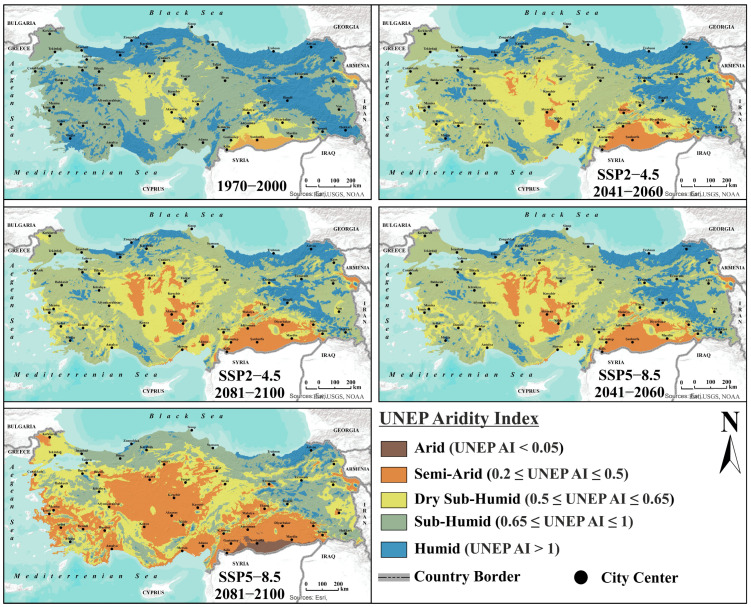
Spatial distribution of the UNEP AI under the reference period and projected future climate scenarios.

Climate projections indicate that under both moderate and pessimistic scenarios, arid and semi-arid areas will expand over time, while sub-humid and humid regions will shrink and become restricted to specific areas. Even under SSP2−4.5 scenario, semi-arid conditions are projected to emerge in Central Anatolia during 2041–2060 and expand further in Southeastern Anatolia. dry sub-humid areas have expanded, particularly in the interior and southern regions, compared to the reference period. The SSP5−8.5 scenario, representing the most pronounced aridification, suggests a dramatic transformation of Türkiye’s climate geography. According to this scenario, semi-arid conditions are projected to dominate the southern regions, Central Anatolia, and the Aegean coastal belt by 2081–2100, with southern semi-arid areas transitioning to fully arid conditions relative to the reference period. In contrast, the Black Sea coast, northern Marmara, and the high-elevation, cooler regions of Eastern Anatolia are expected to retain humid and sub-humid conditions as exceptional regions. Aridification is particularly pronounced in the Mediterranean and Aegean coastal corridors, where traditional olive cultivation takes place (Figure 7).

#### 3.6.1. Is Olive Distribution Shifting Toward Drier Environments?

**Figure 8:**
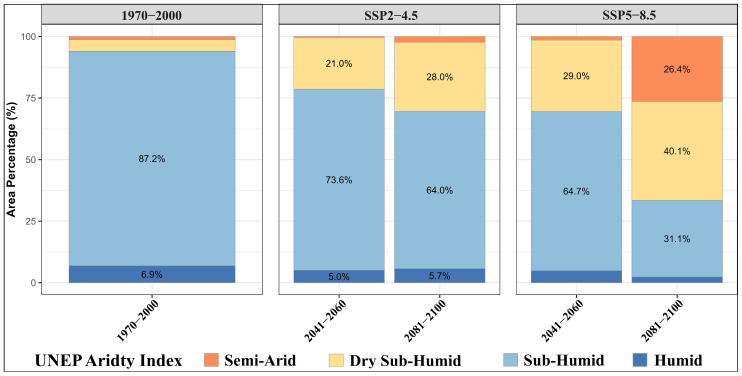
Percentage distribution of suitable olive habitats (highly and moderate suitable classes) across UNEP aridity classes under projected climate scenarios.

The distribution of areas with medium and high suitability for olive cultivation under climate scenarios was analyzed using frequency distributions according to UNEP AI classes. The results indicate that climate change will significantly alter the climatic characteristics of suitable olive habitats, subjecting these areas to increasing aridity stress. During the 1970–2000 reference period, the vast majority (87.2%) of suitable olive habitats were classified as sub-humid, while 6.9% fell within humid conditions (Figure 8). This demonstrates that, under current climatic conditions, olive trees achieve optimal growth primarily in temperate regions with adequate rainfall. However, future scenarios suggest that this balance will be markedly disrupted.

Under the SSP2–4.5 scenario, the proportion of suitable olive habitats in the sub-humid class declines to 73.6%, while dry sub-humid areas increase to 21% during the 2041–2060 reference period. By the end of the century, this trend becomes more pronounced, with sub-humid areas decreasing to 64% and dry sub-humid areas rising to 28%. This indicates that suitable habitats for olive trees are gradually shifting toward drier climatic conditions, and redistributing under increasing water stress. The high-emissions SSP5–8.5 scenario shows an even more dramatic transformation. In the near future, 64.7% of suitable areas fall within the sub-humid class, while 29% are classified as semi-arid. By 2081–2100, 40.1% of suitable areas are projected to be in the semi-arid class and 31.1% in the sub-humid class. Notably, 26.4% of suitable habitats will experience semi-arid conditions during the same period (Figure 8). This finding suggests that by the end of the century, more than two-thirds of olive-suitable areas will be exposed to significant aridity pressure.

#### 3.6.2. Extent of Habitat Loss Attributable to Aridity Conditions

Logistic regression analysis was performed for each climate scenario to statistically assess the extent to which projected future losses in olive habitats are associated with increasing aridity conditions. In this analysis, areas identified as “loss” in the change models were coded as 1, while all other areas were coded as 0 and used as the dependent variable. UNEP AI values for each scenario were used as the independent variable.

The analysis results reveal a highly significant and consistent relationship between increasing aridity and the likelihood of olive habitat loss across all scenarios and periods (Table 6). The *p*-values in all models (<0.001) indicate that this relationship is statistically robust and unlikely to be due to chance. Negative coefficients (β) indicate that the probability of habitat loss rises logarithmically as aridity severity increases (i.e., as AI values decrease). Model performance indicators further support the strength of this relationship. AUC values range from 0.857 to 0.921, suggesting that the models distinguish between areas likely and unlikely to experience habitat loss at a “good” to “excellent” level. Similarly, Pseudo R^2^ values between 31.09% and 49.15% indicate that aridity alone explains a substantial portion of the variance in habitat loss. The magnitude of the effect of aridity is quantitatively reflected in the Odds Ratio (OR) values. Under the moderate-emissions SSP2–4.5 scenario, a one-standard-deviation increase in aridity increases the probability of habitat loss by 6.34 times during 2041–2060 and by 8.74 times by the end of the century. Under the high-emissions SSP5–8.5 scenario, the effect is even more pronounced: a 7.11-fold increase is projected for 2041–2060, rising to 17.71-fold by 2081–2100. These results quantitatively demonstrate that future declines in olive habitats are directly and strongly linked to increasing aridity, with severe aridity under high-emission scenarios exponentially raising the risk of habitat loss.

## 4. Discussion

This study differs from previous research by employing an ensemble approach rather than a single model, using an ensemble of seven GCMs instead of a single global circulation model, and statistically linking suitable habitat classes with habitat losses under projected aridity conditions—the latter representing the most original aspect of the study.

In Türkiye, olives can be economically cultivated at elevations between 0 and 800 m [34]. Above this range, suitable habitats become limited due to changes in temperature regimes, increased frost frequency, and altered humidity conditions. This explains why altitude contributes most to the model: it directly regulates temperature and humidity, as well as the climatic thresholds that define the potential distribution limits of olive trees. Consequently, elevation is one of the key factors shaping the distribution patterns of olive trees in Türkiye [36]. Precipitation, represented by Bio19, is the second most important variable, highlighting the critical role of precipitation, especially during the winter months, in olive ecology.

In regions dominated by the Mediterranean climate, most precipitation occurs during the coldest three months, a period that is critically important for replenishing soil moisture and restoring the plant’s water reserves. Although olive trees are vegetatively dormant in winter, rainfall during this period provides the soil moisture necessary for spring growth, flowering, and fruit set [92]. Therefore, the high contribution of Bio19 is associated with the concentration of the species in coastal and transitional zones, where winter rainfall is sufficient. In contrast, high-altitude and continental areas with limited winter precipitation or increased frost risk show markedly reduced suitability for olive cultivation. The temperature-related variables Bio9, Bio4, and Bio8 represent the thermal ecology of olive trees and their sensitivity to seasonal temperature dynamics. Bio9 reflects physiological resistance to high temperatures and summer aridity, indicating water stress tolerance. Bio4 reveals the impact of annual temperature fluctuations on the species’ phenological cycles and geographic distribution limits. Bio8 characterizes temperature conditions during the wettest period, influencing vegetative growth and shoot development processes. Taken together, these temperature variables show that olive trees achieve optimal distribution in coastal and transitional zones with a temperate climate in Türkiye, whereas suitability declines in inland and high-altitude areas with greater thermal variability.

The findings of this study indicate that the potential distribution of olive trees in Türkiye is primarily determined by the interaction of altitude, winter precipitation, and seasonal temperature dynamics. This explains why the species exhibits high suitability in the Mediterranean, Aegean, and South Marmara coastal belts, while its distribution rapidly declines in high-altitude and continental inland regions. Similar patterns have been reported in the literature regarding the contributions of variables in olive distribution models. For instance, Özdel et al. [38] identified Bio12, Bio7, and Bio9 as the most significant contributors, whereas Özdel et al. [39] found Bio12, Bio4, and Bio9 to be the most influential. Ashraf et al. [93] found that Bio12 and Bio19 contributed significantly to the models they created. In contrast, Yıldız et al. [94] reported that the Bio11, Bio6, and Bio1 variables provided the highest contribution in their study. Differences in variable contribution across studies generally result from variations in climate databases used in species distribution modelling processes (e.g., CHELSA vs. WorldClim), the selection of environmental variables, spatial resolution, study area location, and model parameterization. These elements significantly affect the model’s sensitivity to environmental gradients and the relative importance ranking of variables. In particular, the method for eliminating multicollinearity and the thresholds applied can directly influence model explanatory power and variable contribution coefficients. Additionally, the spatial density and representativeness of occurrence records, along with the ecological diversity of the study area, may introduce sampling bias in the model’s learning process, leading to an increase or decrease in the relative importance of some variables.

The potential olive distribution areas identified for the reference period (1970–2000), which served as the baseline for comparing future suitability models, were found to largely coincide with the current olive cultivation zones. The statistical performance metrics further support this alignment. The high accuracy values (AUC = 0.927; TSS = 0.768) indicate that the model not only performs strongly in classification but also demonstrates high spatial representation. In line with national production statistics [3], the substantial overlap between areas of high and moderate suitability identified by the model and existing production regions confirms that the model accurately represents the actual distribution of olive cultivation. Moreover, the consistency of these results with olive distribution patterns reported in previous studies [34,80,81,94]. Further validates the model’s spatial accuracy and ecological realism. Collectively, these findings demonstrate that the developed model is robust and suitable for evaluating future projections and assessing the potential impacts of climate change on olive cultivation.

The future projections obtained in this study suggest that the potential distribution of olive trees in Türkiye will shift northward and toward higher-elevation inland regions. These spatial shifts are consistent with patterns reported in previous studies conducted both in Türkiye and other geographical regions [37,38,39,49,81,95,96]. Areas with very high suitability are projected to decline under future climate scenarios compared to the reference period, whereas moderate- and low-suitability areas are expected to expand. This trend reflects the northward and altitudinal expansion of the species’ ecological niche, driven by rising temperatures and altered precipitation regimes. In particular, the Black Sea and Marmara regions—previously unsuitable for olive cultivation—are projected to become increasingly favourable for olive production as climatic conditions change. It is important to note that these projected shifts represent conditional outcomes that would occur only if the corresponding SSP emission trajectories materialize, as each scenario reflects different socio-economic pathways and climate forcing. Under these conditions, the resulting ecological shifts can be regarded as ecologically plausible. Moreover, Türkiye is located within the Mediterranean Basin, a well-recognized global climate change hotspot, where pronounced warming trends and significant alterations in temperature and precipitation patterns have already been documented [11,13,18,20,21,23]. These regional climatic dynamics further support the plausibility of the projected shifts in olive suitability under future scenarios.

Studies generally emphasize similar aridity patterns across Türkiye regarding aridity projections although the aridity indices, climate databases, scenarios, and time periods used in the literature vary [19,83,89,97,98,99]. Nevertheless, these methodological differences can lead to partial discrepancies in the reported severity and spatial distribution of aridity in certain local areas. The analyses conducted in this study based on UNEP AI classes corroborate the observed shifts in olive habitat suitability. The findings indicate that, areas with high and moderate suitability for olive cultivation will increasingly transition toward dry sub-humid and semi-arid climate classes in the future. This transition highlights that the disruption of the climatic water balance—driven by rising temperatures and declining precipitation—is placing suitable olive habitats under growing aridity stress. Furthermore, it is anticipated that increased evaporation and reduced precipitation will substantially heighten irrigation demands, threatening the sustainability of rain-fed olive groves [96]. Logistic regression analyses also reveal a statistically significant relationship between UNEP AI values, used as an aridity indicator, and habitat loss. The finding that greater aridity severity increases the likelihood of habitat loss suggests that aridity may be one of the most critical drivers of the projected decline in areas suitable for olive cultivation. Similarly, Arenas-Castro et al. [100] reported that future projections for Andalusia indicate hotter and drier conditions, and that changes in olive suitability areas are closely associated with moisture deficits and reduced rainfall regimes.

Rising temperatures and increasing aridity not only constrain the potential distribution of olive trees but also adversely affect their physiological processes, product quality, and pest–disease dynamics. Climate change is expected to affect olive tree pollen production and flowering timing [101], fruit development [41,102], chilling requirements due to insufficient winter cold [103], and harvest period [104]. These impacts may lead to substantial alterations in both olive yield and oil quality [100,102,104,105,106]. Supporting this concern, Kaniewski et al. [45] empirical evidence from the Levant region, identifying the optimal annual mean temperature range for olive cultivation as 16.9 ± 0.3 °C. Their findings indicate that projected temperature increases in the latter half of the 21st century will likely surpass this threshold, thereby constraining flowering and fruit formation and reducing suitable cultivation areas. Additionally, the study reported that fruit set and oil yield decline markedly when annual precipitation drops below 450 mm.

Changing climatic conditions are also reshaping the regional distribution and severity of olive pests and diseases [107]. In regions where olive cultivation is particularly vulnerable, both the direct and indirect impacts of climate change can lead to declines in product quality and economic value, resulting in income losses for producers. Small-scale enterprises, which often lack sufficient capital and technological capacity, are especially susceptible to these climatic pressures. Consequently, shifts in production patterns driven by climate change may generate substantial socio-economic repercussions, influencing rural income distribution, food security, and agricultural sustainability.

The future of olive ecosystems may be influenced not only by shifts in climatic suitability but also by ecological and socio-economic factors, including agricultural management practices, cultivar selection, efficient water use, the spread of pests and diseases, pressures from urbanization, climate-induced forest fires, and land-use changes. Therefore, mitigating the impacts of climate change on olive production requires a holistic approach, supported by ecologically informed planning, locally tailored adaptation policies, sustainable agricultural practices, and the conservation of natural habitats.

It should be acknowledged that the model results are based solely on climatic suitability, and that increases in temperature and aridity are not the only factors influencing changes in olive habitats. Anthropogenic and biotic drivers—such as urbanization, forest fires, land-use changes, agricultural intensification, shifts toward alternative crops with higher economic returns, and the proliferation of pests and diseases under changing climate conditions—may also contribute substantially to future habitat losses. Within this context, the present study focuses exclusively on climate-based projections, excluding anthropogenic and biotic factors that are difficult to model or predict. This constitutes one of the key limitations of the research. Moreover, the analyses were conducted on *Olea europaea* L., the main species of olive. Considering that more than 400 olive cultivars are found in Türkiye—some restricted to specific regions or even localities, while others are cultivated in mixed forms [34]—the absence or inaccessibility of spatially explicit and detailed data on these cultivars represents another important limitation of the study.

Adaptation strategies for the future are critically important to ensure the sustainability of olive cultivation under changing climatic conditions [38,81]. In the face of increasing aridity and temperature stress, key priorities should include restructuring water management systems, promoting the use of efficient irrigation technologies, adopting agricultural practices that enhance soil organic matter, and developing aridity-tolerant olive cultivars. In parallel, establishing new production infrastructure in areas that were previously unsuitable or partially suitable for olive cultivation in inland and northern regions, strengthening producer organizations, and targeting state support mechanisms will be essential for sustainable adaptation. Implementing these measures will enhance not only environmental sustainability but also social and economic resilience in the face of climate change.

The future of olive production extends far beyond being a purely agricultural concern; it is intrinsically connected to several Sustainable Development Goals (SDGs). Declining production under increasing climate stress poses a threat to SDG 2 (Zero Hunger), while the resulting loss of agricultural income and employment opportunities particularly affects rural communities that depend on agriculture for their livelihoods, directly relating to SDG 8 (Decent Work and Economic Growth). Enhancing the efficiency of olive production, improving water and soil management, and minimizing environmental footprint align with SDG 12 (Responsible Consumption and Production). Furthermore, adaptation measures that mitigate the adverse effects of climate change and reduce greenhouse gas emissions correspond to SDG 13 (Climate Action). The conservation of olive grove ecosystems, maintenance of biodiversity, and prevention of land degradation are also vital for achieving SDG 15 (Life on Land), contributing to both ecological balance and the sustainability of the rural economy. Consequently, olive production should be viewed not merely as an economic activity but as a strategic element that influences the future of the nation’s economy, ecosystem resilience, and social welfare.

## 5. Conclusions

This study examined the effects of climate change on the potential distribution of the olive tree (*Olea europaea* L.) using climate-based projections and species ensemble modelling approaches. The results demonstrate that climate change not only drives olive habitats toward increasingly arid regions but also profoundly alters the climatic and ecological structures that define these habitats. The progressive contraction of the olive tree’s traditional sub-humid distribution zones in the study area indicates that, in the future, habitats are likely to develop a fragmented and heterogeneous configuration—characterized by aridity-adapted semi-arid regions on one side and limited humid refuge areas on the other.

In the reference period, high-suitability areas represented 4.4% of Türkiye. By the end of the century, these areas are projected to decline to 2.6% under the SSP2–4.5 scenario and to only 0.2% under SSP5–8.5. Conversely, moderate-suitability areas—which accounted for 6.4% during the reference period—are expected to increase to 11.6% under SSP2–4.5 and 9.8% under SSP5–8.5 by 2081–2100. Change analysis results show that, relative to the reference period, gains may occur over 10–14%, while losses may range from 8–11% across scenarios.

Under reference conditions, 87.5% of the suitable areas (high + moderate suitability) fall within the sub-humid climatic class. However, projections indicate a significant expansion of dry sub-humid conditions through time. Under SSP2–4.5, dry sub-humid conditions are projected to constitute 21% of suitable areas in 2041–2060 and 28% in 2081–2100. Under the more pessimistic SSP5–8.5 scenario, this climatic class expands even further, reaching 29% in 2041–2060 and 40% in 2081–2100. Additionally, although suitable olive habitats are currently almost absent in semi-arid regions, these areas are expected to expand substantially, accounting for over 26% of suitable areas by the end of the century under SSP5–8.5.

The results indicate that, rather than experiencing a uniform loss of habitat, olive trees are likely to undergo a redistribution pattern polarized between two climatic extremes. The expansion of the semi-arid class suggests that the species may be approaching the upper limits of its ecological tolerance under intensifying aridity stress, while humid regions—particularly high-altitude and coastal areas—may emerge as potential climatic refugia. These findings offer valuable insights into the ecological resilience of olive trees and their adaptive capacity in the face of increasing environmental variability.

Climate change is projected to drive a spatial restructuring of olive production areas, which hold strategic significance for national economy and rural development in Türkiye. In the future, certain regions may partially or entirely lose their suitability for olive cultivation, while areas that previously exhibited limited production potential may become viable under new climatic conditions. This dynamic will create “gaining” and “losing” regions within the olive sector, highlighting the need for a reassessment of regional development policies, agricultural planning, and targeted adaptation strategies.

## Figures and Tables

**Table 1:** Climate Classification and Threshold Values Based on the UNEP Aridity Index (AI).

	Climate Classification	Index Values
**UNEP Aridty Index**	Hyper Arid	UNEP AI < 0.05
	Arid	0.05 ≤ UNEP AI ≤ 0.2
	Semi-Arid	0.2 ≤ UNEP AI ≤ 0.5
	Dry Sub-Humid	0.5 ≤ UNEP AI ≤ 0.65
	Sub Humid	0.65 ≤ UNEP AI ≤ 1
	Humid	UNEP AI > 1

**Table 2:** Algorithms used in the modelling process without applying a threshold value.

Models	AUC	TSS
RF	0.942 *	0.788 *
MaxEnt	0.931 *	0.769 *
BRT	0.931 *	0.767 *
MARS	0.926 *	0.762 *
GAM	0.921 *	0.765 *
SVM	0.914 *	0.757 *
GLM	0.902	0.677
GLMNET	0.896	0.677
CART	0.895	0.699
FDA	0.894	0.683
Mahal.dismo	0.869	0.648
Domain.dismo	0.865	0.625
BIOCLIM	0.706	0.411
Mean	0.927	0.768

*: Indicates the algorithms identified as successful based on AUC and TSS values and subsequently used in the modelling process.

**Table 3:** Contribution values of environmental variables for each algorithm based on high-performing models.

Variables	GAM	Mars	BRT	RF	SVM	MaxEnt	Mean
Elevation	0.32	0.43	0.10	0.06	0.22	0.31	0.24
Bio19	0.18	0.09	0.04	0.04	0.07	0.16	0.10
Bio9	0.06	0.07	0.16	0.07	0.05	0.02	0.07
Bio4	0.03	0.01	0.07	0.04	0.11	0.02	0.05
Bio8	0.08	0.04	0.00	0.01	0.04	0.08	0.04
Bio14	0.04	0.05	0.00	0.01	0.03	0.04	0.03
Bio3	0.00	0.00	0.00	0.01	0.02	0.00	0.01
Slope	0.01	0.01	0.01	0.01	0.02	0.02	0.01
Aspect	0.00	0.00	0.00	0.01	0.01	0.01	0.01

**Table 4:** Quantitative assessment of area (km^2^ and %) occupied by each suitability class for models generated for the reference and projected future periods.

	1970–2000		SSP2–4.5 2041–2060		SSP5.8–5 2041–2060		SSP2–4.5 2081–2100		SSP5–8.5 2081–2100	
Class	km^2^	%	km^2^	%	km^2^	%	km^2^	%	km^2^	%
Unsuitable	606,271	79.8	581,886	76.6	572,557	75.3	563,339	74.1	564,341	74.2
Low Suitability	71,165	9.4	74,371	9.8	81,173	10.7	89,426	11.8	119,138	15.7
Moderate Suitability	48,907	6.4	84,936	11.2	88,824	11.7	87,798	11.6	74,750	9.8
High Suitability	33,723	4.4	18,874	2.5	17,514	2.3	19,504	2.6	1838	0.2

**Table 5:** Quantitative assessment of area (km^2^ and %) changes in gain, loss, and stable suitability categories for future projections relative to the reference period.

	SSP2−4.5 2041–2060		SSP5−8.5 2041–2060		SSP2−4.5 2081–2100		SSP5−8.5 2081–2100	
Change	km^2^	%	km^2^	%	km^2^	%	km^2^	%
Unsuitable	551,025	72.5	539,566	71.0	527,432	69.4	527,976	69.5
Stable	66,431	8.7	59,533	7.8	54,227	7.1	52,875	7.0
Gain	81,862	10.8	95,834	12.6	109,910	14.5	93,692	12.3
Loss	60,748	8.0	65,134	8.6	68,498	9.0	85,524	11.3

**Table 6:** Logistic regression analysis of habitat loss based on UNEP Aridity Index and change models.

Scenario	Period	Coefficient (β)	*p*-Value	Odds Ratio (OR)	Pseudo R^2^	AUC
SSP2−4.5	2041–2060	−1.8463	<0.001	6.34	31.09	0.857
SSP2−4.5	2081–2100	−2.1675	<0.001	8.74	38.92	0.888
SSP5−8.5	2041–2060	−1.9616	<0.001	7.11	34.31	0.870
SSP5−8.5	2081–2100	−2.8739	<0.001	17.71	49.15	0.921

## Data Availability

All data used in this study were obtained from publicly available sources. The bioclimatic variables, temperature, and precipitation data for the 1970–2000 reference period were downloaded from the WorldClim database (https://www.worldclim.org/data/worldclim21.html). Future scenario data, including bioclimatic variables, temperature, and precipitation, were also obtained from the same WorldClim database https://www.worldclim.org/data/cmip6/cmip6_clim30s.html. Elevation, slope, and aspect maps were derived from the SRTM dataset provided by WorldClim, which is available at https://www.worldclim.org/data/worldclim21.html. Species occurrence records were downloaded from the Global Biodiversity Information Facility (GBIF) open-access database (https://www.gbif.org/occurrence/download/0254332-230224095556074). All analyses were performed using the open-source R software (version 4.4.1) (https://www.r-project.org/), and species distribution modelling was conducted with the sdm package https://cran.r-project.org/web/packages/sdm/index.html. The datasets generated and/or analyzed during the current study are available from the corresponding author upon reasonable request.

## Supplementary Materials

The following supporting information can be downloaded at: https://www.mdpi.com/article/10.3390/plants14243774/s1, Figure S1: Bioclimatic variables.
